# Rice Husk Silica Liquid Enhances Autophagy and Reduces Overactive Immune Responses via TLR-7 Signaling in Lupus-Prone Models

**DOI:** 10.3390/ijms251810133

**Published:** 2024-09-21

**Authors:** Chieh Kao, Shih-Wei Wang, Po-Chun Chen, Chun-Yung Huang, Yu-Feng Wei, Cheng-Hsun Ho, Yong-Han Hong

**Affiliations:** 1 Department of Medical Laboratory Science, College of Medical Science and Technology, I-Shou University, Kaohsiung 82445, Taiwan; ekingo@gmail.com (C.K.); chenghsunho@gmail.com (C.-H.H.); 2 School of Medicine, College of Medicine, I-Shou University, Kaohsiung 82445, Taiwan; ed101397@edah.org.tw (S.-W.W.); yufeng528@gmail.com (Y.-F.W.); 3 Division of Allergy, Immunology and Rheumatology, Department of Internal Medicine, E-Da Hospital, I-Shou University, Kaohsiung 82445, Taiwan; 4 Department of Life Science, National Taiwan Normal University, Taipei 116059, Taiwan; pcchen@ntnu.edu.tw; 5 Department of Seafood Science, National Kaohsiung University of Science and Technology, Kaohsiung 81157, Taiwan; cyhuang@nkust.edu.tw; 6 Department of Internal Medicine, E-Da Cancer Hospital, Kaohsiung 82445, Taiwan; 7 Graduate Programs of Nutrition Science, National Taiwan Normal University, Taipei 116059, Taiwan

**Keywords:** systemic lupus erythematosus, rice husk silica liquid, toll-like receptor 7, autophagy, inflammation, TNF-α

## Abstract

Systemic lupus erythematosus (SLE) is a chronic autoimmune disorder characterized by widespread inflammation and multi-organ damage. Toll-like receptor 7 (TLR-7) and autophagy have been implicated in SLE pathogenesis. Rice husk silica liquid (RHSL) has shown potential for modulating inflammatory responses, but its effects on SLE have not been thoroughly investigated. This study aims to evaluate the impact of RHSL on immune responses and autophagy in cell culture experiments, focusing on its effects on TLR-7 signaling, cytokine production, and autophagy modulation. RAW264.7 cells and human peripheral blood mononuclear cells (PBMCs) from healthy donors and SLE patients were used. Cells were stimulated with LPS or TLR-7 agonists and treated with RHSL. Cell viability was assessed, and cytokine levels (TNF-α and IL-6) were measured by ELISA. Autophagy-related proteins (LC3II, ATG5-ATG12) were analyzed by Western blotting. The effect of autophagy inhibition was studied using 3-methyladenine (3-MA). A concentration of 100 μg/mL RHSL did not affect cell viability but significantly reduced the TNF-α production in TLR-7 agonist-stimulated RAW264.7 cells (compared to TLR-7 alone, 3.41 ± 0.54 vs. 6.72 ± 0.07 folds) and PBMCs (compared to TLR-7 alone, 0.97 ± 0.19 vs. 1.40 ± 0.33 folds). RHSL enhanced autophagy, as evidenced by increased LC3II (4.35 ± 1.08 folds) and ATG5-ATG12 (7.07 ± 1.30 folds) conjugation in both RAW264.7 cells and SLE patient-derived PBMCs. The reduction in TNF-α production by RHSL was attenuated by 3-MA, indicating that autophagy plays a role in this process. RHSL also inhibited the translocation of phosphorylated NF-κB into the nucleus, suggesting a mechanism for its anti-inflammatory effects. RHSL exhibits potential as an immunomodulatory agent in SLE by enhancing autophagy and modulating TLR-7 signaling pathways. These findings suggest that RHSL could offer therapeutic benefits for managing inflammatory responses in SLE and warrant further investigation into its clinical applications.

## 1. Introduction

Immune-mediated inflammatory diseases (IMIDs) such as rheumatoid arthritis (RA), systemic lupus erythematosus (SLE), inflammatory bowel disease (IBD), psoriasis, multiple sclerosis (MS), and type 1 diabetes (T1D) show diverse global incidence [1,2]. RA affects around 1.3 million people in the U.S., with higher prevalence in developed countries. Psoriasis is also more common in developed regions. IBD rates are increasing globally, including in developing countries [2]. SLE impacts about 72.8 in 100,000 in the U.S., with a higher prevalence among women and certain ethnic groups [3]. Understanding the epidemiology and clinical features of these diseases is essential for developing effective treatments and improving patient outcomes.

Systemic lupus erythematosus (SLE) is a chronic autoimmune disease characterized by widespread inflammation and damage to multiple organ systems, including the skin, joints, brain, lungs, and kidneys. Patients with SLE frequently present with a range of symptoms such as mucosal damage, arthritis, and manifestations affecting the neurological and psychiatric domains, including headaches, seizures, acute confusion, and cognitive dysfunction [4,5]. Among SLE complications, lupus nephritis is particularly concerning, affecting over 40% of patients, with 10–20% of these progressing to chronic kidney disease. According to the European League Against Rheumatism (EULAR), SLE management should focus on reducing disease activity and preventing relapses [6]. Treatment typically involves glucocorticoids combined with immunosuppressants. However, glucocorticoids can cause weight gain, hypertension, and osteoporosis, while hydroxychloroquine may lead to gastrointestinal issues and retinopathy, with both medications carrying rare but serious side effects.

SLE arises from a complex interplay of genetic factors, environmental influences, and disturbances in immune regulation [7]. Central to its pathogenesis are not only immune complexes formed by autoantibodies but also various components of the innate immune system. Toll-like receptors (TLRs), a subset of pattern-recognition receptors (PRRs), play a crucial role in detecting pathogen-associated molecular patterns (PAMPs) and initiating innate immune responses [8]. A growing body of research indicates that TLR signaling plays a crucial role in SLE development [9]. Among the TLRs, TLR-7, an endosomal receptor, is notably expressed in macrophages, plasmacytoid dendritic cells, and some B cell subtypes [10]. Activation of TLR-7 with imiquimod exacerbates lupus nephritis in MRL-*lpr*/*lpr* mice [11]. TLR-7 is implicated in the pathogenesis of lupus nephritis in both murine and human studies by recognizing endogenous ribonucleoprotein antigens, thus activating antigen-presenting cells [12]. A recent study indicated that enhanced TLR-7 signaling promotes aberrant survival of B cell receptor (BCR)-activated B cells, and gain-of-function genetic variations in TLR-7 can cause human lupus [13].

In addition, dysregulated autophagy contributes to increased inflammation and inappropriate cell death, both of which are central features in lupus pathology [14]. Autophagy facilitates the clearance of cellular debris, including apoptotic cells [15]. The impaired clearance of apoptotic cells can result in the presentation of self-antigens and the subsequent development of autoantibodies, a hallmark of lupus. TLRs regulate autophagy through various mechanisms. For instance, TLR-4 activation induces autophagy via MyD88 and TRIF-dependent pathways [16]. Conversely, TLR-7 and TLR-8 activation in cancer cells promotes cell survival. In lung cancer cells, TLR-7 stimulation upregulates Bcl-2 protein expression, thereby inhibiting autophagy and enhancing cell survival [17]. Furthermore, TLR-7 activation reduces autophagy by suppressing miR-15b, leading to increased cyclin D3 (CCND3) expression in B cells, which is associated with autoimmune conditions such as SLE [18]. When TLR-7 is activated in macrophages, it can influence the polarization and function of these cells. TLR7 activation has been shown to enhance the production of pro-inflammatory cytokines and chemokines, amplifying the inflammatory response mediated by M1 macrophages [19]. A previous study demonstrated that paeonol, when administrated in TLR-7/8 agonist-induced lupus models, can suppress the polarization of macrophages to the M1 and promote their polarization to the M2. This effect is associated with the inhibition of MAPK and NF-κB signaling pathways, leading to the amelioration of lupus nephritis [20]. Such interactions are significant in conditions like lupus nephritis, where immune activation and inflammation are central to the disease pathology.

Silicon (Si) is known as an essential component of collagen and glycosaminoglycan formed in bones and cartilage. A high silica (SiO_2_) content was found in rice husk, which contains approximately 17~20% ash that consist of mainly silica content (>90%). Previous studies demonstrated that silica nanoparticles from rice husk are more biocompatible [21], thus expecting more applications in the food industry. Our previous studies demonstrated that rice husk silica liquid (RHSL), administered in STZ-induced diabetic models, enhances autophagy in pancreatic islet cells and mitigates cell apoptosis induced by a reactive oxygen species (ROS) [22,23]. Exploring the bioactive properties of RHSL under pathological conditions is highly promising. However, to date, no research has investigated the potential role of RHSL in SLE. Therefore, this study aims to elucidate the relationship between autophagy and the immunomodulatory effects of RHSL in SLE.

## 2. Results

### 2.1. Effects of RHSL, LPS and TLR-7 Agonist on RAW264.7 Cell Viability

**Figure 1:**
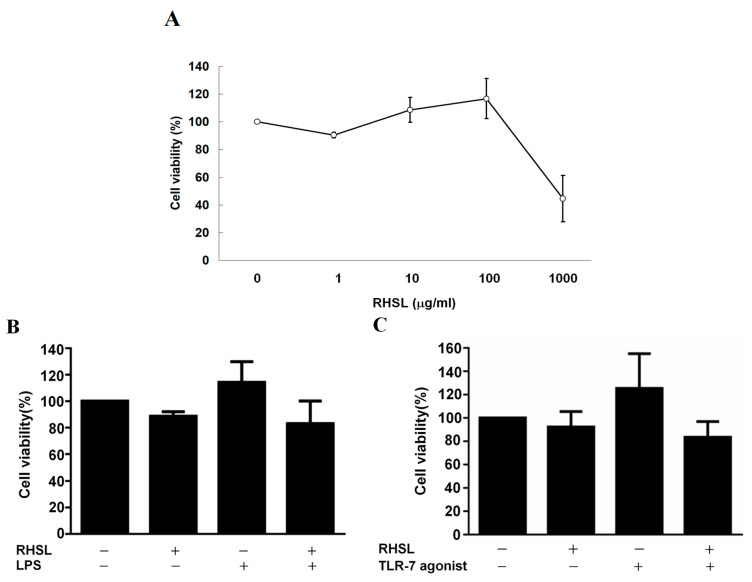
The effect of RHSL treatment on RAW264.7 cell viability. RAW 264.7 cells were treated with either vehicle alone or RHSL at the indicated concentrations (1, 10, 100, 1000 µg/mL) for 24 h, and cell viability was assessed using the MTT assay (**A**). Additionally, cell viability was evaluated following treatment with 100 µg/mL RHSL in the absence or presence of 2 µg/mL LPS (**B**) or 10 µg/mL TLR-7 agonist (**C**) for 24 h. Data are representative of at least three independent experiments.

In this study, TLR agonists and LPS are used to induce overactive immune responses in vitro, thereby simulating the immune conditions associated with SLE. This investigation aims to determine whether RHSL is capable of mitigating this overactive immune response and further elucidate the mechanisms through which RHSL triggers its immunomodulatory effects. RAW264.7 cells served as the in vitro model. Results showed that there was no effect on cell viability at a 1:100 dilution of RHSL (100 μg/mL) (Figure 1A). RHSL at a concentration of 100 μg/mL were used in the following experiments. LPS and the TLR-7 agonist were used on RAW264.7 cells to induce an overactive immune response. Our data demonstrated that the treatments (LPS or TLR-7 agonist alone, or combined with RHSL, respectively) did not influence cell viability compared to the control group after 24 h of culture (Figure 1B,C).

### 2.2. RHSL Suppresses LPS or TLR Agonist-Induced TNF-α Secretion

**Figure 2:**
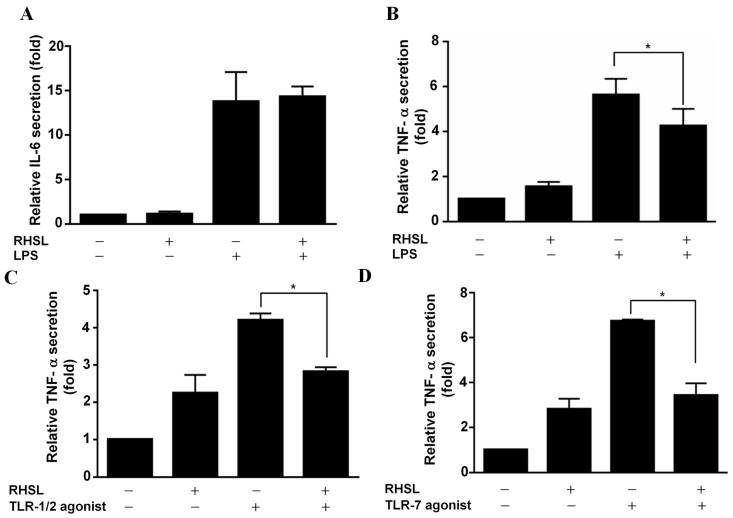
Effects of RHSL on cytokine secretion in LPS- or TLR agonist-stimulated RAW264.7 cells. Cells were treated with LPS or RHSL, either alone or in combination, for 24 h. IL-6 secretion in the supernatants was measured using an ELISA (**A**). TNF-α secretion was measured by ELISA in supernatants from cells treated with LPS (**B**), TLR-1/2 agonist (**C**), TLR-7 agonist (**D**), or RHSL, either alone or with the indicated stimulators, for 24 h. Data are representative of at least three independent experiments and are expressed as means ± SD. Statistical significance was determined using two-tailed *t*-tests with SPSS statistical software. A line with a star symbol connecting two bars indicates a significant difference between those groups (* *p* < 0.05).

To elucidate the effect of RHSL on the modulation of inflammation, the secretion levels of two important cytokines, IL-6 and TNF-α, were analyzed by ELISA. The data show that both cytokines were significantly increased in response to LPS, indicating that LPS did stimulate and activate RAW264.7 cells (Figure 2). However, when RAW264.7 cells were stimulated with LPS in the presence of RHSL treatment, the secretion levels of TNF-α were significantly reduced (Figure 2B), while IL-6 levels were not affected by RHSL (Figure 2A). Next, our study used TLR-1/2 or TLR-7 agonists as inducers to stimulate RAW264.7 cells. As expected, treatment with either the TLR-1/2 agonist (Figure 2C) or the TLR-7 (Figure 2D) agonist significantly increased the secreted levels of TNF-α in RAW264.7 cells. However, this increase in TNF-α levels in response to the TLR-1/2 agonist or the TLR-7 agonist was decreased by RHSL treatment. The above results suggest that RHSL may play a regulatory role in modulating the overactive immune response of SLE by reducing cytokine secretion.

### 2.3. RHSL Induces Autophagy in LPS or TLR Agonist-Induced RAW264.7

**Figure 3:**
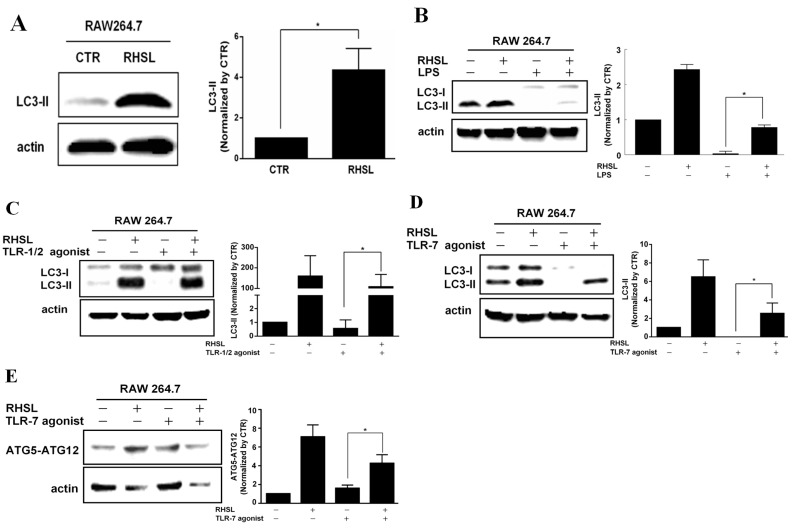
Effects of RHSL on the expression of autophagy-related proteins in LPS- or TLR agonist-stimulated RAW264.7 cells. Cells were treated with a concentration of 100 μg/mL RHSL for 24 h. LC3 protein expression in cell lysates was assessed by Western blotting (**A**). LC3 expression was also evaluated in cell lysates from cells treated with a concentration of 2 μg/mL LPS (**B**), 1 μg/mL TLR-1/2 agonist (**C**), 10 μg/mL TLR-7 agonist (**D**), or 100 μg/mL RHSL, either alone or in combination with the indicated stimulators, for 24 h. Additionally, ATG5-ATG12 conjugate expression was assessed in cell lysates from cells treated with a concentration of 10 μg/mL TLR-7 agonist (**E**) or 100 μg/mL RHSL, either alone or in combination, for 24 h. Quantitative data from band intensities are representative of at least three independent experiments and are expressed as means ± SD. Statistical significance was determined using two-tailed *t*-tests with SPSS statistical software. A line with a star symbol connecting two bars indicates a significant difference between those groups (* *p* < 0.05).

According to previous studies, it was hypothesized that autophagy plays a crucial role in immunomodulatory effects. To investigate whether RHSL modulation correlates with autophagy in an SLE-associated context characterized by overactive immune responses, we assessed autophagy-related proteins. The results showed that the protein expression of LC3II decreased in response to both LPS and TLR agonists in RAW264.7 cells. In contrast, treatment with RHSL alone (Figure 3A) or in combination with LPS or TLR agonists (Figure 3B–D) significantly enhanced LC3II protein expression. A similar trend was observed for the expression of ATG5-ATG12 conjugation; RHSL treatment alone or in combination with LPS or TLR agonists increased the expression of ATG5-ATG12 conjugation in RAW264.7 cells (Figure 3E). Furthermore, LPS or TLR agonists alone decreased the expression of LC3-II significantly. Collectively, these results suggest that RHSL induces autophagy and counteracts the reduced autophagy induced by LPS or TLR agonists in RAW264.7 cells.

### 2.4. RHSL Decrease Secretion of TNF-α via Induction of Autophagy and Inhibition of Translocation of NF-kB in RAW264.7

**Figure 4:**
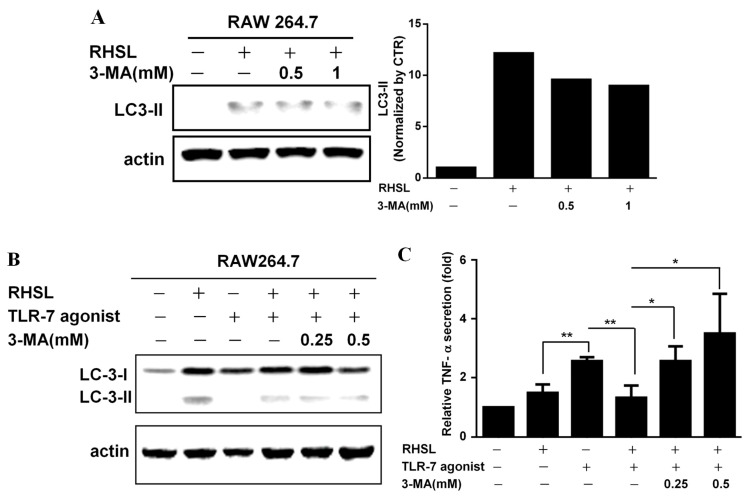
Interactions of RHSL with LC3II expression and TNF-α secretion in TLR-7 agonist-stimulated RAW264.7 cells. Cells were treated with a concentration of 100 μg/mL RHSL in the absence or presence of 0.5 or 1 mM 3-MA for 24 h. LC3II protein expression in cell lysates was assayed by Western blotting (**A**). LC3I/II expression (**B**) and TNF-α secretion in supernatants (**C**) were evaluated in cells treated with a concentration of 10 μg/mL TLR-7 agonist and 100 μg/mL RHSL, with or without 0.25 or 0.5 mM 3-MA for 24 h. Quantitative data are representative of at least three independent experiments and are expressed as means ± SD. Statistical significance was determined using two-tailed *t*-tests with SPSS statistical software. A line with a symbol, such as single star (* *p* < 0.05) or double stars (** *p* < 0.01), connecting two bars indicates a significant difference between those groups.

**Figure 5:**
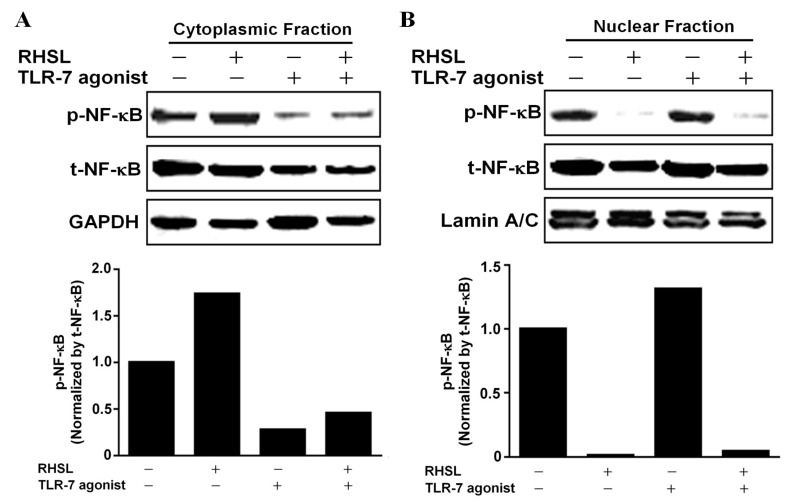
Effects of RHSL on the translocation of phosphorylated NF-κB into the nucleus in TLR-7 agonist-stimulated RAW264.7 cells. Cells were treated with a concentration of 10 μg/mL TLR-7 agonist or 100 μg/mL RHSL, either alone or in combination, for 24 h. Nuclear-cytoplasmic fractionation analysis was performed to quantify the levels of phosphorylated NF-κB and total NF-κB in cytoplasmic (**A**) and nuclear (**B**) protein extracts, respectively. GAPDH and Lamin A/C were used as internal controls for cytoplasmic and nuclear fractions, respectively.

We then investigated whether RHSL-induced autophagy could mitigate the over-immune response triggered by LPS or TLR agonists. 3-MA (3-methyladenine) is an autophagic inhibitor (Figure 4A). Treatment with 3-MA increased TNF-α secretion by suppressing RHSL-induced autophagy, suggesting that RHSL-induced autophagy could alleviate the immune response in this in vitro model (Figure 4B,C). The TLR-7 agonist or LPS primarily stimulate TNF-α secretion by phosphorylating NF-κB, which subsequently translocate into the nucleus to initiate transcription. Our findings indicate that RHSL can reduce TNF-α secretion induced by TLR-7 agonist or LPS; however, the mechanisms underlying RHSL’s reduction in TNF-α secretion are not yet clear. Next, to explore the changes in NF-κB phosphorylation in RAW264.7 cells treated with TLR-7 agonist and RHSL, nuclear-cytoplasmic fractionation was performed to analyze NF-κB translocation. The results revealed RHSL treatment alone notably reduced the nuclear phosphorylated NF-κB (Figure 5B). Moreover, RHSL effectively attenuated TLR-7 agonist-induced phosphorylation and nuclear translocation of NF-κB (Figure 5). These findings clearly indicate that RHSL inhibits the translocation of phosphorylated NF-κB, thereby blocking TNF-α secretion induced by TLR-7 agonist.

### 2.5. RHSL Activates Autophagy and Adjusts Level of Secretions of Cytokines in Human Peripheral Blood Mononuclear Cells (PBMC) from Healthy Donors and SLE Patients.

**Figure 6:**
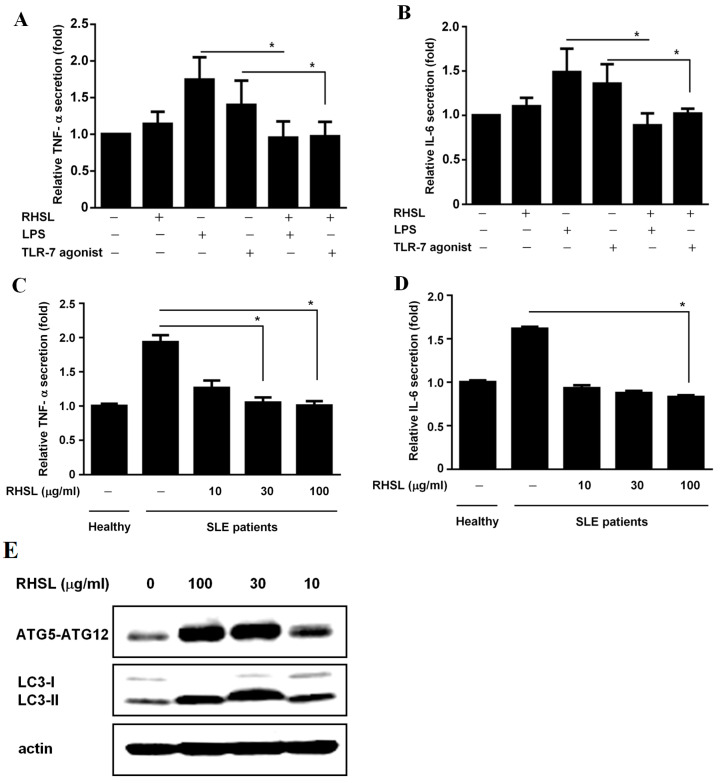
Interactions of RHSL with LC3II expression and cytokine secretion in PBMCs from SLE patients. PBMCs from healthy donors were treated with concentrations of 2 μg/mL LPS, 10 μg/mL TLR-7 agonist, or 100 μg/mL RHSL, either alone or with the indicated stimulators for 24 h. TNF-α (**A**) and IL-6 (**B**) secretion in the supernatants were measured by ELISA. PBMCs from SLE patients were treated with either the vehicle or RHSL at concentrations of 10, 30, or 100 μg/mL for 24 h. Supernatants were then collected and analyzed for TNF-α (**C**) and IL-6 (**D**) secretion by ELISA. Cell lysates from these treatments were examined for LC3I/II protein expression and ATG5-ATG12 conjugate level (**E**). Data are representative of at least four independent experiments and are expressed as means ± SD. Statistical significance was determined using two-tailed *t*-tests with SPSS statistical software. A line with a star symbol connecting two bars indicates a significant difference between those groups (* *p* < 0.05).

To investigate the clinical associations between cytokine secretion and SLE, we collected the peripheral blood mononuclear cells (PBMCs) from both healthy donors and SLE patients. We then examined whether RHSL can modulate cytokine secretion in these PBMCs. The results showed that RHSL significantly reduced the secretion of IL-6 and TNF-α stimulated by either LPS or TLR-7 agonist in PBMCs from healthy donors (Figure 6A,B). Additionally, the study assessed the dose-dependent effects of RHSL on cytokine secretion in PBMCs. Consistent with our hypothesis that an aberrant cytokine profile is present in SLE, we observed that PBMCs from SLE patients secrete higher levels of TNF-α and IL-6 spontaneously compared to PBMCs from healthy individuals. Notably, RHSL reduced the secretion levels of IL-6 and TNF-α in PBMCs from SLE patients in a dose-dependent manner, with significant effects observed particularly at a concentration of 100 μg/mL (Figure 6C,D). Furthermore, RHSL also increased the levels of ATG5-ATG12 conjugation and LC3-II in PBMCs from SLE patients in a dose-dependent manner, with significant increases noted at concentrations of 30 and 100 μg/mL (Figure 6E).

## 3. Discussion

Rice husk silica liquid (RHSL) is derived from rice husk silica (RHS), a high-purity silicon dioxide. RHSL was prepared by dissolving RHS in alkaline solutions. The silicon concentration in RHSL was precisely quantified using inductively coupled plasma optical emission spectroscopy (ICP-OES), ensuring an accurate dosage for subsequent experiments. Our initial experiments established that RHSL concentrations ≤ 100 μg/mL were non-toxic to RAW264.7 cells. This is consistent with a prior study by Kim et al. [24], which demonstrated that sodium silicate at a concentration of 1000 μM (28 μg/mL silicon) did not adversely affect cell viability over a 3-day period, whereas 10,000 μM (280 μg/mL silicon) significantly reduced it. Our findings confirm that the silicon concentrations used in our study are within safe limits and suitable for further investigation.

Silicon is essential for the synthesis of collagen and glycosaminoglycans, which are critical for maintaining healthy bones and cartilage [25]. Beyond these structural roles, silicon exhibits bioactive properties, including antioxidant and anti-inflammatory effects. For example, research has demonstrated that silicon at a concentration of 100 μM enhances radical scavenging and reduces inflammatory markers such as TNF-α, iNOS, and COX-2 in LPS-induced RAW264.7 cells [24]. Similarly, our results showed that RHSL, at a concentration of 100 μg/mL, reduces TNF-α production in both LPS-induced and TLR-7 agonist-induced RAW264.7 cells, as well as in human peripheral blood mononuclear cells (PBMCs). Clinical research further supports the role of silicon in managing oxidative stress and regulating inflammatory processes in rheumatoid arthritis patients [26]. However, the precise mechanisms through which silicon or RHSL modulates inflammation remain underexplored, highlighting an important area for further research.

To address this gap, we investigated the effects of RHSL on autophagy and TLR-7 signaling, with a particular focus on their relevance to the overactive immune responses observed in lupus. TLR-7 gain-of-function mutations are recognized as a monogenic cause of human lupus [8], and hyperactive TLR-7 signaling is a known driver of autoimmune diseases in mouse models. Recent studies used TLR-7 agonists, such as imiquimod (IMQ) or R848, to induce lupus-like conditions in mice, assessing various treatments’ effects on SLE-related pathologies, including hypertension [27] and lupus nephritis [20]. In our experiments, the referenced studies indicate that a concentration of 10 μg/mL IMQ is more effective in enhancing the immune response [28,29]. It was employed as a TLR-7 agonist to induce pro-inflammatory cytokine production and the translocation of phosphorylated NF-κB into the nucleus in RAW264.7 cells (Figure 5) and PBMCs from healthy donors (Figure 6). Our study is among the first to demonstrate that RHSL-induced autophagy blocks the nuclear translocation of NF-κB. However, the specific molecular targets and the interaction with the TLR-7 pathway remain unclear. Previous studies showed that an autophagy induction can lead to NF-κB inactivation [30,31]. The precise mechanism is not fully understood, and this will be a key focus of our future research.

Regarding autophagy, our results showed that LPS, the TLR-1/2 agonist, or the TLR-7 agonist generally reduced the expression of autophagy-related proteins. This is consistent with previous studies where LPS decreased LC3II expression in RAW264.7 macrophages in a dose-dependent manner (10–100 ng/mL) [32]. In line with our results, IMQ was shown to reduce levels of autophagy-related proteins, including phosphorylated ULK-1, ATG-7, and LC3A/B, in murine models of psoriasis-like inflammation, [33,34]. However, some studies reported contrasting effects of LPS or TLR agonists on autophagy [35,36,37,38]. For instance, Lee et al. [37] observed increased LC3II protein expression in RAW264.7 cells stimulated with IMQ at a concentration of 2 μg/mL, while our study utilized IMQ at a concentration of 10 μg/mL for 24 h. Shang et al. [38] noted that autophagy-related responses peaked at 12 h after LPS stimulation and then declined. These discrepancies suggest that autophagy may dynamically modulate inflammatory signaling at different stages of inflammation.

Wu and Lu [39] highlighted the crucial role of autophagy in macrophage polarization and function, affecting the balance between pro-inflammatory (M1) and anti-inflammatory (M2) macrophages. Despite not observing an increase in autophagy-related proteins in response to stimulators in our experiments, we detected an elevated production of pro-inflammatory markers, indicating that RHSL may influence inflammatory pathways through mechanisms beyond autophagy alone. Interestingly, the treatment with RHSL increased the expression levels of these autophagy-related proteins and reduced TNF-α production in LPS, the TLR-1/2 agonist, or TLR-7 agonist-stimulated macrophage cell lines (Figure 2 and Figure 3). Notably, the effect of RHSL treatment on enhancing autophagy and reducing TNF-α production was diminished when co-treated with 3-MA, an autophagy inhibitor. Chloroquine is a commonly used medication for patients with systemic lupus erythematosus (SLE), based on the supplementary data (Supplementary Figure S1), comparison of RHSL and chloroquine (CQ), RHSL alleviate TLR-7 agonist-induced TNF-α more effectively. This suggests that autophagy modulation is closely linked to the regulation of inflammatory responses and highlights RHSL’s significant role in promoting autophagy, which in turn influences inflammation. Some current studies indicate that TLR-7 agonists elevate immune responses by decreasing autophagy [33,34]. However, the underlying mechanism remains not fully understood. This is an important aspect that we aim to clarify in our future research.

In evaluating the effects of RHSL on immune responses, we used PBMCs from both healthy donors and SLE patients. This approach provides a more physiologically relevant model compared to RAW264.7 cells. Our results demonstrate that RHSL effectively attenuates TNF-α and IL-6 production in PBMCs from healthy donors in response to LPS or TLR-7 stimulation (Figure 6A,B). In contrast, RHSL did not significantly reduce IL-6 production in the RAW264.7 cell line (Figure 6C,D). This discrepancy may be due to the heterogeneous cellular composition of PBMCs, which includes monocytes, T lymphocytes, and B lymphocytes, whereas RAW264.7 cells are a homogeneous macrophage-like cell line that does not fully replicate the complex immune interactions observed in PBMCs. Based on the SLE patients PBMCs data, it reveals the dose-dependent effects of RHSL, moreover, supplementary results (Supplementary Figure S2) revealed 100 μg/ml RHSL performed the most effectively to trigger autophagy.

It is known that TLR-7 functions as a sensor of viral RNA and binds to guanosine [8]. Brown et al. [13] suggested that a deficiency of MyD88, an adaptor protein downstream of TLR-7, could mitigate autoimmunity such as aberrant B cell survival and serological phenotypes, indicating that gain-of-function genetic variations in TLR-7 can cause human lupus. We hypothesize that RHSL may modulate IL-6 production by affecting either lymphocytes or monocytes. Compared to healthy donors, PBMCs from SLE patients exhibit elevated TNF-α and IL-6 levels (Figure 6). Notably, the RHSL treatment resulted in a significant reduction in the production of these cytokines in SLE patients-derived PBMCs. Meanwhile, PBMCs from SLE patients displayed lower expression levels of the autophagy-related proteins ATG-5 and LC3II, which increased in a dose-dependent manner following RHSL treatment (Figure 6E). These observations suggest RHSL modulates aberrant autophagy and inflammatory responses in SLE-derived primary cells.

## 4. Materials and Methods

### 4.1. Rice Husk Silica Liquid (RHSL) Preparation and Reagents

Rice husk was obtained from a local rice miller and was soaked in a 3% aqueous organic acid solution containing citrate and tartaric acid to remove lignin and other crude fibers. The treated rice husk was then washed three times with distilled water. It was subsequently subjected to smoldering at 300 °C for 2 h, followed by combustion at 800 °C for 2 h to yield rice husk silica (RHS). The resulting RHS was dissolved in a NaOH solution to prepare a 2% (*w*/*v*) aqueous RHS suspension, referred to as RHS liquid (RHSL). This RHSL suspension was then diluted with a culture medium to achieve the desired concentration and used as the test sample in the subsequent experiments. The reagents used in this study included 3-methyladenine (3-MA, Sigma-Aldrich, St. Louis, MO, USA), lipopolysaccharides (LPS, MedChemExpress, Monmouth Junction, NJ, USA), toll-like receptor (TLR)-7 agonists (imiquimod, MedChemExpress, Monmouth Junction, NJ, USA) and TLR1/2 agonists (Novus Biologicals, Centennial, CO, USA). To ensure stable storage of appropriate concentrations, the 3-MA and TLR-7 agonist (imiquimod) were dissolved in dimethyl sulfoxide (DMSO) to achieve concentrations of 266 mM and 5 mg/L, respectively. LPS and TLR1/2 agonists were dissolved in sterile water to concentrations of 1 mg/mL and 5 mg/L, respectively. These stimulator reagents were diluted into the appropriate concentrations using corresponding culture media for subsequent experimental use.

### 4.2. RAW264.7 Cell Culture and Viability Measurement

The murine cell line RAW264.7 (Bioresource Collection and Research Center, Hsinchu, Taiwan; BCRC60001) was cultured in Dulbecco’s modified Eagle’s medium (DMEM)/F12, supplemented with a 10% heat-inactivated fetal bovine serum (FBS) in a humidified incubator at 37 °C with 5% CO_2_. While conducting the experiments, the cells were seeded on either a 96-well plate (cell density of 1 × 10^4^ cells/well) or 6 cm plate (cell density of 7 × 10^5^ cells/well, 50–60% confluency) for the indicated assays. The cells were treated with either the vehicle media or the indicated concentration of RHSL (30, 50, or 100 μg/mL) in the presence of the stimulator. After a 24 h culture, culture supernatants were collected and the cells in the bottom of the plates were examined for their viability by using the 3-(4,5-dimethylthiazol-2-yl)-2,5-diphenyltetrazolium bromide (MTT) method, which is followed from our previous study [40]. Briefly, 55 μL of DMEM/F12 medium containing 1 mg/mL of MTT was loaded onto the plate and incubated for 3 h. Then, 100 μL of isopropanol containing 0.04 N HCl was added to each well to dissolve the colorful crystals. The cell viability was determined at the absorbance of 570 nm using an automated scanning multiple-well spectrophotometer (SPECTROstar Nano, BMG LABTECH, Ortenberg, Germany). Collected culture supernatants were analyzed for the cytokines production. Additionally, cell lysates from indicated plates were harvested to detect the expressions of the target proteins.

### 4.3. Blood Samples from Healthy Donors and SLE Patients

Blood samples were collected to isolate peripheral blood mononuclear cells (PBMCs). Systemic lupus erythematosus (SLE) patients were recruited based on a diagnosis of SLE according to the 1982 American College of Rheumatology (ACR) criteria. This study included four healthy donors and nine SLE patients, all over 20 years of age. The SLE patients had disease activity index scores (SLEDAI) ranging from 5 to 14. The inclusion criteria for SLE patients included regular use of anti-inflammatory drugs or immunosuppressants, complete medication records, no medication administration within 18 h prior to blood collection, and non-pregnant status. These criteria were adapted from Lin et al. [41]. This study was approved by the Human Research Ethics Committee at E-DA Hospital (EMRP-110-184/202109RHSL), and all participants provided informed consent prior to enrollment.

### 4.4. Isolation and Culture of Peripheral Blood Mononuclear Cells (PBMCs)

PBMCs were isolated from whole blood using density gradient centrifugation. Blood samples were collected in heparinized tubes and diluted 1:1 with phosphate-buffered saline (PBS). The diluted blood was carefully layered onto a Lymphoprep™ density gradient medium (STEMCELL Technologies, Cologne, Germany) at a 2:1 ratio (volume of diluted blood to volume of Lymphoprep™). The mixture was centrifuged at 800× *g* for 20 min at room temperature without break to maintain gradient integrity. PBMCs were collected from the interface between the plasma and the Lymphoprep™ medium. The PBMC layer was aspirated, transferred to a new sterile tube, and washed with PBS. Following a 300× *g* centrifugation for 10 min at room temperature, the supernatant was discarded, and the PBMC pellet was resuspended in DMEM/F12 containing 10% fetal bovine serum (FBS; STEMCELL Technologies, Germany). The cells were counted and adjusted to the desired concentration for further experiments. PBMCs at the density of 5 × 10^5^ cells/well (50–60% confluency) were cultured in DMEM/F-12 medium supplemented with 10% FBS in 6-well plates for 24 h. After the initial culture period, PBMCs were treated with varying concentrations of RHSL for an additional 24 h. Supernatants were collected for immediate testing or stored at −20 °C for cytokine analysis. Cells adhering to the bottom of the plates were reserved for subsequent protein measurements.

### 4.5. Cytokine Production Assay

The cytokine secretion was measured using an enzyme-linked immunosorbent assay (ELISA). The assays were conducted with the ELISA MAXTM Deluxe Set for human/mouse IL-6 and TNF-α (Cat.no. 431304 and Cat.no. 430904, limits of quantification is 7.8 pg/mL, Biolegend, San Diego, CA, USA), following the manufacturer’s instructions, capture antibodies 100 μL/well were incubated on 96-well flat-bottom plates at 4 °C overnight. After incubation, the solution was removed and the wells were washed with a washing buffer. The wells were then blocked with assay diluent 200 μL/well for 1 h at room temperature (RT). Sample supernatants were diluted with assay dilute as needed and added to the wells (200 μL/well) for 2 h at RT. Following this, at RT, detection antibodies 100 μL/well were added and incubated for 1 h, followed by the addition of 100 μL/well avidin-HRP conjugate and incubation for an additional 30 min. Plates were washed at least twice with washing buffer between each step. Finally, wells were developed with 100 μL/well TMB substrate for 20–30 min, causing a color change that corresponds to the amount of cytokine present. The reaction was stopped with sulfuric acid, and optical density was measured at 450 nm using an automated scanning multiwell spectrophotometer (SPECTROstar Nano, BMG LABTECH, Ortenberg, Germany).

### 4.6. Subcellular Fractionation

To obtain nuclear extracts, we used a commercial Nuclear Extraction Kit (NBP2-29447, Novus Biologicals, Centennial, CO, USA). Cells were washed with cold phosphate-buffered saline (PBS) containing phenylmethylsulfonyl fluoride (PMSF) and then resuspended and lysed using a hypotonic buffer supplemented with a detergent solution. The whole cell lysate was centrifuged at 14,000 rpm for 10 min at 4 °C. The supernatant, which contains the cytosolic fraction, was carefully collected. The nuclear pellet was resuspended in a nuclear extraction buffer on ice and homogenized. This suspension was then centrifuged at 14,000 rpm for another 10 min at 4 °C. The supernatant, containing the nuclear fraction, was collected for further analysis. Proteins from both cytosolic and nuclear fractions were analyzed for total and phosphorylated NF-κB by using Western blotting.

### 4.7. Western Blotting Analysis

Western blotting was performed according to the protocol adapted from Kao et al. [42]. Briefly, cells were lysed in ice-cold RIPA lysis buffer containing 1% Triton X-100, 1% NP-40, 0.1% SDS, 0.5% deoxycholate (DOC), 20 mM Tris–HCl (pH 7.4), 150 mM NaCl, and a mixture of protease and phosphatase inhibitors (Sigma) for 30 min. Protein separation was carried out using 10% and 13% SDS-PAGE resolving gels. Proteins were transferred to nitrocellulose membranes (PALL, Show Low, AZ, USA) and blocked with BlockPRO Protein-Free Blocking Buffer (Visual protein, Taipei City, Taiwan) for 30 min. The membranes were probed with primary antibodies: anti-LC3 (1:5000, Novus Biologicals, USA; NB100-2331), anti-ATG5 (1:5000, Novus Biologicals, USA; NB110-53818), anti-NF-κB p65 (1:4000, Novus Biologicals, USA; NB100-56712SS), and anti-phospho-NF-κB p65 (Ser536) (1:1000, Novus Biologicals, USA; NB100-82088). After incubation with corresponding horseradish peroxidase-conjugated secondary antibodies (Jackson ImmunoResearch Laboratories, West Grove, PA, USA), proteins were visualized using LumiFlash™ Ultima Chemiluminescent Substrate (Visual Protein, Taipei City, Taiwan). The band intensities were quantified and normalized to β-actin (20536-1-AP, Proteintech, Rosemont, IL, USA).

### 4.8. Statistical Analysis

All data are expressed as mean ± standard error of the mean (SEM), unless otherwise specified. The results are representative of at least three independent experiments. Group comparisons were performed by using two-tailed *t*-tests with SPSS statistical software (Version 17; SPSS Inc., Chicago, IL, USA). A two-tailed *p*-value of less than 0.05 was considered statistically significant.

## 5. Conclusions

**Figure 7:**
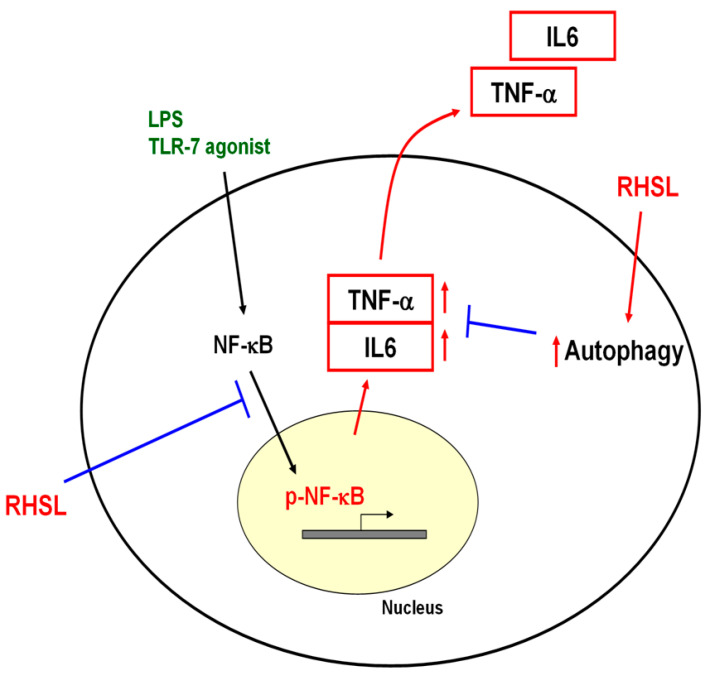
Proposed mechanism of RHSL action in the TLR-7 signaling pathway. RHSL is hypothesized to reduce TNF-α secretion by enhancing autophagy and interfering with the translocation of phosphorylated NF-kB into the nucleus within the TLR-7 signaling pathway.

In summary, this study elucidates the mechanism by which RHSL, a soluble form of silicon, influences overactive immune responses. Specifically, RHSL enhances autophagy through modulation of TLR-7 signaling, disrupting the translocation of phosphorylated NF-κB into the nucleus and leading to reduced TNF-α production (Figure 7). These findings provide new insights into how RHSL could potentially modulate inflammatory responses and offer a foundation for future research into therapeutic applications.

## Data Availability

The data can be available upon request.

## Supplementary Materials

The following supporting information can be downloaded at: https://www.mdpi.com/article/10.3390/ijms251810133/s1.
