# Supplementary material for: Rice Husk Silica Liquid Enhances Autophagy and Reduces Overactive Immune Responses via TLR-7 Signaling in Lupus-Prone Models
Source: Int J Mol Sci. 2024 Sep 21;25(18):10133. doi: 10.3390/ijms251810133 (PMC11432151; doi:10.3390/ijms251810133)
Supplement: Supplementary file 1 [file ijms-25-10133-s001.zip › ijms-3198426-supplementary.pdf]

Supplementary Figure S1

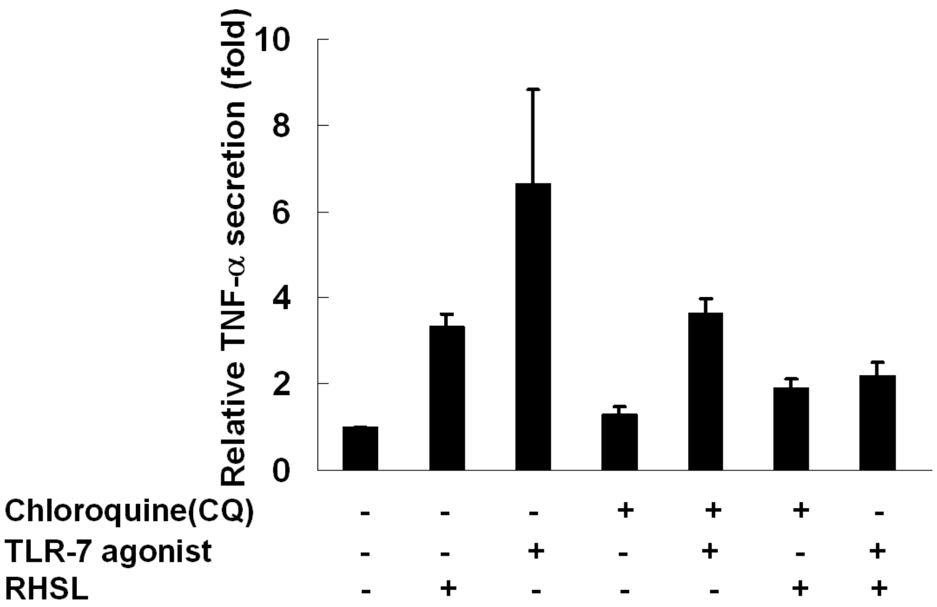

Supplementary Figure S1. TNF-α secretion in supernatants were evaluated in cells treated with 10 g/mL TLR-7 agonist and 100 μg/mL RHSL, with or without 140 M chloroquine (CQ) for 24 hours. Quantitative data are representative of at least three independent experiments and are expressed as means ± SD. Statistical significance was determined using two-tailed *t*-tests with SPSS statistical software. A line with a symbol, such as single star ($p < 0.05$) or double stars ($p < 0.01$), connecting two bars indicates a significant difference between those groups.

Supplementary Figure S2

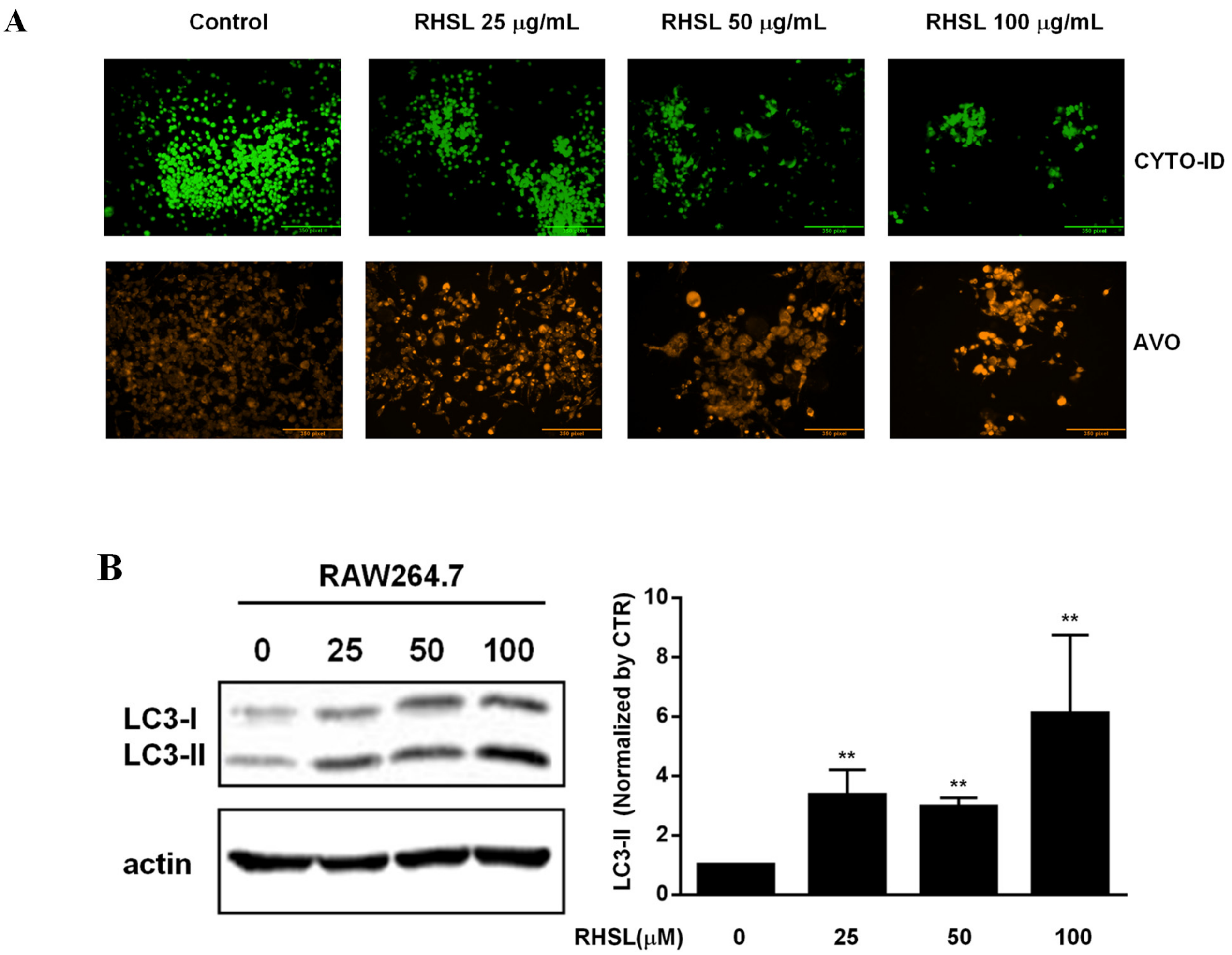

Supplementary Figure S2. (A) Top panel: Treatment with RHSL (25, 50, 100 μg/ml) increased the formation of fluorescent LC3 puncta in RAW264.7 cells in a dose-dependent manner. LC3 puncta was detected by CYTO-ID. Bottom panel: The numbers of autolysosome were increased by RHSL in a dose-dependent manner. Acidic Organelle vesicles (AVO) was detected by Acridine orange. (B) Cell lysates from treatments of RHSL (25, 50, 100 μg/ml) were examined for LC3I/II protein expression. Data are representative of at least four independent experiments and are expressed as means ± SD. Statistical significance was determined using two-tailed *t*-tests with SPSS statistical software. A line with a star symbol connecting two bars indicates a significant difference between those groups ($p < 0.05$).
